# Domain-Specific Computational, Functional and Structural Methods Enable Interpretation of *BRCA1* BRCT Variants of Uncertain Significance

**DOI:** 10.3390/curroncol33060354

**Published:** 2026-06-11

**Authors:** Gabriella C. Torretto, Matthew D. Martin, Kaamraan Islam, Nicole E. Archer, Harriet E. Feilotter, Scott K. Davey

**Affiliations:** 1 Department of Pathology and Molecular Medicine, Queen’s University, Kingston, ON K7L 3N6, Canada; 17gct@queensu.ca (G.C.T.);; 2 Division of Cancer Biology and Genetics, Sinclair Cancer Research Institute, Queen’s University, Kingston, ON K7L 3N6, Canada; 3 Laboratory Medicine Program, University Health Network, Toronto, ON M5G 2C4, Canada

**Keywords:** *BRCA1*, hereditary breast cancer, hereditary ovarian cancer, variants of uncertain significance, germline genetic testing, in silico, functional assays, structural modeling

## Abstract

Background: Pathogenic germline *BRCA1* and *BRCA2* variants cause most hereditary breast and ovarian cancers. Widespread genetic testing has revealed thousands of variants with unknown effects on disease risk, known as variants of uncertain significance (VUS). *BRCA* VUS, the majority of which are missense, complicate genetic test interpretation and clinical decision-making. This study aimed to evaluate *BRCA1* VUS pathogenicity with enhanced accuracy through computational, functional and structural methods. Methods: We characterized the structural distribution of *BRCA1* variants. In silico tools scored known consequence variants within a specific region of *BRCA1*. The Molecular Feature Selection Tool (MolecularFeaST; Renwick Lab at Queen’s University; Kingston, ON, Canada) performed feature selection of the most discriminative tools. MATLAB (MATLAB R2024a; Mathworks; Natick, MA, USA) Classification Learner Application trained supervised machine learning models using combinations of the most accurate tools; the best model assigned pathogenicity prediction scores to VUS. Select VUS were functionally assessed through phosphopeptide binding pull-down assays and structurally analyzed on PyMOL (v2.4.1; Schrödinger Inc.; New York, NY, USA). Results: The RING and BRCT domains were identified as hotspots for missense pathogenic variants and VUS; BRCT was selected as the focus of the computational classifier. Nine in silico tools (CADD hg19, MetaRNN, ClinPred, VEST4, BayesDel AD, EVE, Eigen PC, gMVP and PolyPhen2) defined the BRCT-specific missense variant classifier. Twenty-two VUS (R1699P, F1704S, W1837L, W1712G, F1734S, V1804A, I1674V, V1804L, V1804I, I1807V, T1675S, I1764L, N1774I, E1698K, Q1848K, P1749S, A1669T, N1774H, L1839V, T1658I, L1705I, V1654L) demonstrated varying phosphopeptide binding ability and protein levels relative to the wildtype. Computational structural modeling contextualized VUS phosphopeptide interactions and structural implications. Conclusions: We provide in silico and functional evidence for the classification of *BRCA1* BRCT VUS and highlight the utility of domain-specific computational approaches for characterizing missense variants in multi-domain genes.

## Simple Summary

Most hereditary breast and ovarian cancers are caused by pathogenic germline variants in the tumor suppressor genes *BRCA1* and *BRCA2*. Widespread genetic testing has revealed thousands of variants with unknown effects on disease risk, known as variants of uncertain significance (VUS). VUS complicate genetic test interpretation and subsequent screening, prophylaxis and treatment decisions. In populations enriched for genetic predisposition to the development of breast and ovarian cancers, we identified *BRCA1*’s RING and BRCT domains as VUS hotspots. In this study, we developed a computational classifier tailored to the BRCT domain to predict the pathogenicity of all VUS within this region. Functional assays and structural modeling were performed on twenty-two VUS with scores spanning the classifier’s scale. Our study provides in silico and functional evidence for the classification of *BRCA1* VUS and highlights the utility of domain-specific computational approaches for characterizing missense variants.

## 1. Introduction

Approximately 5–10% of breast and 20–25% of ovarian cancers are inherited [1,2]. Most hereditary cases occur in the context of hereditary breast and ovarian cancer (HBOC) syndrome, an autosomal dominantly inherited cancer predisposition associated largely with *BRCA1* and *BRCA2* pathogenic variants [3,4]. Pathogenic variant carriers have a significantly elevated lifetime risk of developing breast (45–60%) and ovarian cancer (25–40%) [5]. Germline genetic testing is used to detect pathogenic variants in at-risk individuals, informing clinical decision-making for personalized care including enhanced screening, preventative strategies, targeted therapies and cascade testing for at-risk family members [6,7,8,9,10]. Genetic tests can also reveal no sequence alterations or the presence of benign variants, indicating no elevated risk of *BRCA1/2*-associated cancers [11]. The widespread uptake of clinical genetic testing has led to a significant increase in identification of variants of uncertain significance (VUS) [12]. VUS are DNA sequence alterations with uncertain effects on gene function and disease risk [13]. Between 10 and 20% of *BRCA1/2* genetic screens report VUS, with missense variants accounting for over 50% of identified VUS [14]. VUS pose a critical clinical challenge as genetic tests with VUS cannot be effectively interpreted and complicate risk assessment processes [15].

*BRCA1* is a pleiotropic tumor suppressor gene composed of functionally diverse structural domains. The N-terminal RING (amino acids 1–109) and C-terminal BRCT (amino acids 1646–1855) domain are *BRCA1*’s most essential and highly conserved domains, each playing direct roles in *BRCA1*-mediated genomic maintenance [10]. The RING domain is primarily involved in *BRCA1*’s E3 ubiquitin ligase activity [16]. The BRCT domain forms macro-complexes with key phosphopeptide binding partners, Abraxas, CtIP and BACH1, to facilitate DNA damage signaling and mediate different stages of homologous recombination [17]. BRCT’s tandem repeats, BRCT1 and BRCT2, form a binding cleft that recognizes the phosphopeptides’ phosphorylated peptide motif, consisting of a phosphorylated serine and phenylalanine anchor separated by any two residues (pSer-X-X-Phe) [18]. Their statuses as functional domains within *BRCA1* have made the RING and BRCT domains the focus of numerous targeted studies aimed at identifying deleterious variants, reflected by the elevated density of pathogenic variants in these regions.

The ACMG-AMP standards and guidelines for the interpretation of sequence variants, developed by the American College of Medical Genetics and Genomics (ACMG) and Association for Molecular Pathology (AMP), are the most widely used system for interpreting the pathogenicity of genetic variants [19,20]. These guidelines recommend the classification of variants based on weighted criteria (supporting < moderate < strong < very strong < stand-alone) comprising different evidence types, including population, computational, functional, cosegregation, de novo and allelic data [20]. Variants classified as VUS either lack sufficient evidence to support a classification or have conflicting evidence [15]. Missense variants account for the majority of *BRCA1* VUS as there is often insufficient clinical and experimental evidence to achieve a benign or pathogenic classification. The very low minor-allele frequency of individual missense variants and their typical lack of gross structural or functional changes limits the application of cosegregation, population and functional analyses [21,22]. According to the guidelines, VUS should not be used in clinical decision-making, and VUS carriers should receive the same management as probands with no *BRCA1/2* variants [20]. Computational/in silico predictive programs can predict the damaging effect of missense and other variants based on different aspects of sequence variation, including evolutionary conservation, biochemical and structural contexts, protein function and variant allele frequency [20,23]. Several *BRCA1*-specific functional assays, including homology-directed repair (HDR) and binding assays, assess the impact of missense variants on different aspects of its structure and established mechanisms [24,25].

With pathogenic *BRCA1* variants associating with increased risk of breast and ovarian cancers, accurate identification of pathogenic variant carriers is critical. The increasing number of *BRCA1* VUS highlights the need for robust methods to obtain evidence for variant classification, particularly for missense variants. In this study, we characterize the structural distribution of missense VUS in *BRCA1* to discern its critical regions of interest. Through a tailored computational variant classifier, functional pull-down assays and structural modeling, we predict the pathogenicity of VUS within a specific region of *BRCA1* with enhanced accuracy as well as contextualize the molecular mechanisms by which VUS may impact *BRCA1* tumor suppressive function. Our findings are expected to provide in silico and functional evidence for variant classification, as well as insight into the efficacy of domain-specific models in predicting variant pathogenicity in pleiotropic genes.

## 2. Materials and Methods

### 2.1. BRCA1 Variant Distribution Graphing

All *BRCA1* missense variants were collected from the ClinVar database (National Center for Biotechnology Information; Bethesda, Rockville, MD, USA) [26]. Variants were divided into 3 groups based on ClinVar classifications: pathogenic (pathogenic and likely pathogenic), benign (benign and likely benign) and VUS (VUS, conflicting interpretations and variants where no classification was provided). Missense mutations were grouped into sequential 20-amino acid intervals spanning the full length of *BRCA1*, with resulting bins being plotted as histograms to visualize the distribution of variant density across *BRCA1*.

### 2.2. Model Building and Application

All missense variants spanning the BRCT domain (amino acids 1650–1850) were divided into 3 groups based on their ClinVar classifications: pathogenic (pathogenic and likely pathogenic), benign (benign and likely benign) and VUS (VUS, conflicting interpretations and variants where no classification was provided). The in silico tools assembled for this study were identified through the ACMG/AMP standards and guidelines in silico tool table and literature reviews. No tools were excluded based on perceived performance or assumptions regarding relevance to *BRCA1*. The variants in the pathogenic and benign groups were scored by all tools, with each tool’s raw scores scaled on a 0–1 scale (0 = benign and 1 = pathogenic). The pathogenic and benign variants were divided into training and test sets using an 80:20 ratio, with each set consisting of variants spanning the whole BRCT domain. Feature selection was completed using the Molecular Feature Selection Tool (MolecularFeaST; Renwick Lab at Queen’s University; Kingston, ON, Canada), an ensemble-type machine learning application that uses a greedy method based on the ensemble results of 14 feature selection algorithms to rank features by discriminatory ability [27]. With all algorithms enabled with 5-fold cross-validation, MolecularFeaST ranked in silico tools by their discriminatory ability on the training set. The MATLAB Classification Learner Application (MATLAB R2024a; Mathworks; Natick, MA, USA) was used to train multiple supervised machine learning models on training set data, with 5-fold cross-validation. Multiple training sets were employed, starting with the most discriminative in silico tool ranked by MolecularFeaST, and sequentially adding the next highest-ranked tool. Automated training was performed across all 32 available supervised models. Models were built by sequentially incorporating additional tools until the accuracy of the top-performing model declined, serving as the cut-off point. The model with the highest overall accuracy on both the training and test sets was selected to classify the VUS. Binomial 95% confidence intervals for accuracy, sensitivity and specificity were calculated using the Wilson score method. Scaled scores for the VUS group were only obtained from the selected in silico tools integrated into the chosen classifier. The classifier assigned prediction scores to each VUS. Select VUS were chosen for functional analysis, spanning from the benign to the pathogenic extreme of the score scale. Known pathogenic and benign variants from the training set with documented functional evidence from the literature were selected as controls for functional analyses.

### 2.3. DNA Fragment Design, Cloning and Ligation

Double-stranded gene fragments (Integrated DNA Technologies; Coralville, IA, USA) of the *BRCA1* BRCT domain were designed for the wildtype, pathogenic, benign and selected VUS sequences. Fragments were 753 base pairs in length, consisting of flanking *Bam*HI and *Not*I restriction enzyme sites, and an N-terminal 1× FLAG tag fused in frame to the BRCT coding region (nucleotides 4894-5592). DNA fragments were cloned into pCR-Blunt II-TOPO plasmids using the Zero Blunt TOPO PCR Cloning Kit (Invitrogen; Burlington, ON, Canada). Constructs were transformed into One Shot TOP10 Chemically Competent *E. coli* (Invitrogen) and incubated on warmed lysogeny broth (LB) + kanamycin (50 μg/mL) selective agar plates at 37 °C overnight. Select colonies were cultured in 5 mL of LB + kanamycin medium at 37 °C overnight with agitation. Constructs were isolated using the QIAprep Spin Miniprep Kit (QIAGEN; Louisville, KY, USA). Constructs were enzymatically digested with *Bam*HI and *Not*I restriction enzymes (New England Biolabs; Whitby, ON, Canada) in rCutSmart buffer (New England Biolabs) for 25 min and separated by gel electrophoresis in 1.2% agarose gels at 180 V. BRCT fragments were excised as agarose slices and purified using the QIAquick Gel Extraction Kit (QIAGEN). Fragments were ligated into the *Bam*HI and *Not*I cut sites of the pTRE2 mammalian expression plasmid (6241-1, Clontech, Takara Bio USA, Inc.; San Jose, CA, USA) using T4 DNA Ligase (M0202) (New England Biolabs). The custom pTRE2-BRCT constructs were transformed into One Shot TOP10 Chemically Competent *E. coli* and incubated on warmed LB + ampicillin (100 μg/mL) selective agar plates at 37 °C overnight. Select colonies were cultured in 5 mL of LB + ampicillin medium overnight at 37 °C with agitation. Constructs were isolated using the QIAprep Spin Miniprep Kit (QIAGEN). Diagnostic digestion with BamHI/NotI/MscI restriction enzymes and Sanger sequencing (The Centre for Applied Genomics Sequencing Facility; Toronto, ON, Canada) confirmed successful cloning. Constructs were amplified and isolated using the GeneJET Plasmid Maxiprep Kit (Thermo Fisher Scientific; Burlington, ON, Canada).

### 2.4. Cell Culture and Transfection

The Human Embryonic Kidney 293T (HEK293T; ATCC#: CRL-1573; RRID: CVCL_0063; obtained from Queen’s University Sinclair Cancer Research Institute, Kingston, ON, Canada) cell line was used for all cell culture experiments. Cell culture medium consisted of high-glucose, 1% Na-pyruvate Dulbecco’s Modified Eagle Medium (Gibco, Thermo Fisher Scientific) supplemented with 10% fetal bovine serum (FBS; Gibco, Thermo Fisher Scientific) and 1% penicillin/streptomycin (Gibco, Thermo Fisher Scientific). Cell cultures were maintained at 37 °C in a 5% CO_2_ atmosphere humidified incubator. For each variant, 25 μg of pTRE2-BRCT constructs were transfected in duplicate 10 cm plates with a density of 3 × 10^6^ cells/mL using Lipofectamine 3000 Transfection Reagent (Invitrogen). Following a 48 h incubation, cells were harvested via trypsinization and lysed with Pierce IP Lysis Buffer (Thermo Fisher Scientific) substituted with 1% 100× Halt Protease Inhibitor Cocktail (Thermo Fisher Scientific) per million cells. Cell lysate protein concentration was measured using the Pierce BCA Protein Assay Kit (Thermo Fisher Scientific). Three biological replicates of cell culture and lysate collection were conducted for each control and VUS.

### 2.5. Co-Immunoprecipitation and Immunoblotting

Cell lysates were co-immunoprecipitated using Pierce anti-DYKDDDDK (anti-FLAG) Magnetic Agarose (Thermo Fisher Scientific), in which the magnetic agarose slurry was scaled to protein concentrations. BRCT proteins were eluted using 1× sodium dodecyl sulfate (SDS) + 2% β-mercaptoethanol (BME) sample buffer. A total of 5 µg of co-immunoprecipitated lysates were loaded in 4–20% Mini-PROTEAN TGX Precast Protein Gels (Bio-Rad; Mississauga, ON, Canada) and subjected to gel electrophoresis in 1× SDS running buffer at 120 V. Proteins were transferred from gels to nitrocellulose membranes using a Semi-Dry Transfer Apparatus (Bio-Rad) at 20 V for 45 min. Due to its larger molecular weight, BACH1 was transferred with a CriterionTM Blotter (Bio-Rad) at 120 V and 0.3 A at 4 °C overnight. Membranes were blocked with 1× phosphate-buffered saline with 0.1% Tween-20 (PBS-T) in 5% skim milk for 1 h. Membranes were incubated with FLAG (F7425, Sigma-Aldrich; Oakville, ON, Canada), Abraxas (ab139191, Abcam; Waltham, MA, USA), CtIP (9201S, Cell Signaling Technology; Whitby, ON, Canada) and BACH1 (4578S, Cell Signaling Technology) primary antibodies diluted in 1× PBS-T with 1% skim milk at 4 °C overnight. Membranes were washed with 1× PBS-T for 30 min, changing the buffer every 5 min. Membranes were incubated with goat anti-rabbit secondary antibody conjugated to IRDye 680RD (926-68071, LI-CORbio; Lincoln, NE, USA) for 1 h in the dark. Membranes were imaged using the Odyssey DLx Infrared Imaging System (LI-CORbio), with signals quantified using Image Studio (LI-CORbio). Relative FLAG and phosphopeptide signals were calculated by dividing signal intensities across controls, references and VUS by their corresponding wildtype control signal intensities. Immunoblot signals were collected in biological triplicate for each variant. One-tailed one-sample t-tests were performed to compare relative VUS, pathogenic control and benign reference signal intensities against the wildtype control, set to 1. Tests assumed independence of biological replicates. All analyses were conducted in GraphPad Prism Version 10.2.2 (Dotmatics; La Jolla, CA, USA). A significance threshold of *p* < 0.05 was applied.

### 2.6. Flow Cytometry

The Label IT Tracker Intracellular Nucleic Acid Localization Kit with Cy5 fluorophore (MIR 7020) (Mirus Bio; Burlington, ON, Canada) was used to label pTRE2-BRCT wildtype and pathogenic control constructs. Labeled constructs were transfected in 12-well plates seeded the day prior with 1 × 10^6^ HEK293T cells. Non-transfected HEK293T cells were used as the control. After a 48 h incubation, cells were harvested via trypsinization, resuspended in cold phosphate-buffered saline with 1% FBS at a concentration of 1 × 10^5^ cells/mL, and strained into conical tubes. Cells were flowed through the CytoFLEX 10 flow cytometer (Beckman Coulter; Brea, CA, USA), in which cells were characterized using a red laser (638 nm) configured to the CytoFLEX allophycocyanin (APC) channel. Gates were applied to the Forward Scatter Area vs. Side Scatter Area and Forward Scatter Area vs. Forward Scatter Height plots. Signals detected from fluorescence emitted from cells successfully transfected with Cy5 fluorophore-labeled plasmids were graphed on a univariate histogram depicting fluorescent signaling intensity (APC-A) vs. cell count.

### 2.7. Anti-BRCA1 Co-Immunoprecipitation and Immunoblotting

The Dynabeads Antibody Coupling Kit (Invitrogen) was used to couple an anti-BRCA1 antibody (AF6955, R&D Systems; Minneapolis, MN, USA) specific to the *BRCA1* BRCT domain to Dynabeads magnetic beads. Control and VUS pTRE2-BRCT constructs were transfected in HEK293T cells, incubated, harvested and lysed, following the same cell culture and transfection protocols previously outlined. Co-immunoprecipitation of the lysates were conducted using the anti-BRCA1-coupled Dynabeads magnetic beads. Immunoblots were conducted on the co-immunoprecipitated samples, in which membranes were probed with FLAG, Abraxas and CtIP primary antibodies, as previously outlined. Subsequent immunoblot steps followed the same immunoblotting protocol previously outlined.

### 2.8. Computational Structural Modeling

Three-dimensional structural data of BRCA1 BRCT bound to Abraxas, CtIP and BACH1 were obtained through Protein Data Bank (PDB) (National Institutes of Health; Piscataway, NJ, USA) [28]. The structural context of each selected VUS within BRCT was visualized in PyMOL (v2.4.1; Schrödinger Inc.; New York, NY, USA) [29]. Wildtype residues of VUS that interact directly with one or multiple phosphopeptides were substituted with corresponding VUS residues using PyMOL Wizard to visualize their impact on phosphopeptide binding.

## 3. Results

### 3.1. The RING and BRCT Domains Are Hotspots for Missense Pathogenic Variants and VUS

**Figure 1:**
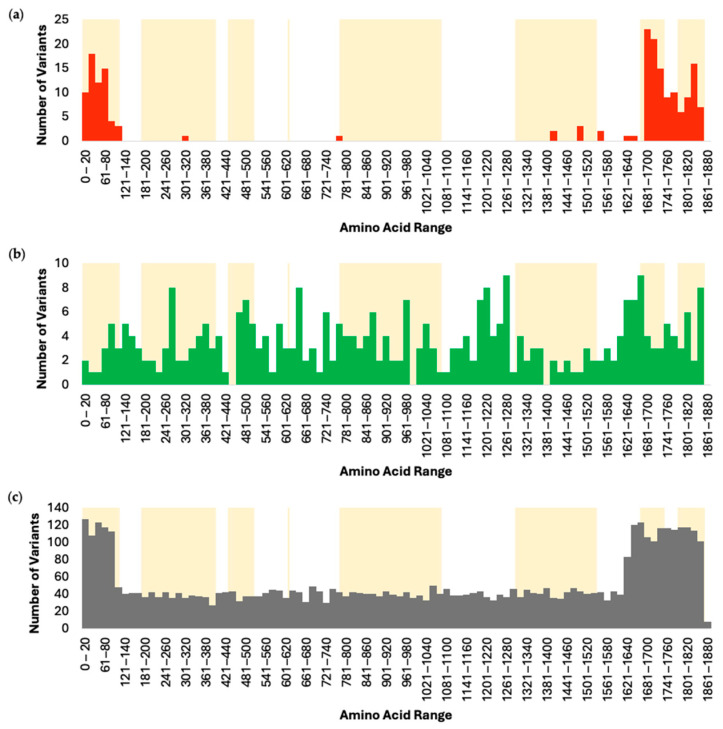
*BRCA1* missense variant distribution across *BRCA1*. Distribution of *BRCA1* missense variants submitted to ClinVar as (**a**) pathogenic or likely pathogenic, (**b**) benign or likely benign, and (**c**) VUS, conflicting interpretations, and variants where no classification was provided displayed in histograms. 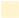 Functional domain and binding site regions.

*BRCA1* missense variant distribution histograms were generated for the pathogenic (*n* = 191), benign (*n* = 348), and VUS (*n* = 4873) variant groups, obtained from ClinVar February 2024 (Figure 1). Pathogenic variants clustered at the RING (~33% of variants) and BRCT domains (~61% of variants) while benign variants appeared to distribute more evenly across the gene. The VUS histogram displayed a clustering pattern resembling that of pathogenic variants. The elevated density of VUS to the BRCT domain led to its selection as the focus of the computational classifier. Although the RING domain was also identified as a key region of interest, its major structural and functional differences from BRCT placed it beyond the scope of this work and will be addressed in future study.

### 3.2. Nine In Silico Tools Define BRCT-Specific Missense Variant Pathogenicity Predictor Tool

BRCT missense variants were placed into pathogenic (*n* = 115), benign (*n* = 53) and VUS (*n* = 1194) variant groups. The VUS group consisted of VUS (*n* = 283), variants for which no classification was provided (*n* = 764) and conflicting interpretations (conflicting benign *n* = 97, conflicting pathogenic *n* = 47). Of the 50 in silico tools assembled, 39 were from the dbNSFP database on the Ensembl Variant Effect Predictor (VEP) web interface [30,31]. The training set comprised 92 pathogenic and 43 benign groups variants, and the test set comprised 23 pathogenic and 10 benign group variants. MolecularFeaST ranked the 50 in silico tools by discriminatory ability and generated a predictive plot showing the rates of decline in predictive performance as the tools descended in order of importance. (Supplementary Table S1, Supplementary Figure S1). Classifiers were incrementally trained on the MATLAB Classification Learner Application up to the top 13th MolecularFeaST-ranked tool, as predictive performance of the most accurate models declined following the 9th ranked tool (Supplementary Table S2). An Ensemble Subspace k-nearest neighbors (kNN) classifier, trained with the top nine in silico tools (CADD hg19, MetaRNN, ClinPred, VEST4, BayesDel AD, EVE, Eigen PC, gMVP and PolyPhen2) demonstrated the best overall performance, achieving 91.1% accuracy (95% CI: 85.1–94.8%) and 87.9% accuracy (95% CI: 72.7–95.2%) on the training set’s validation fold and the test set, respectively. This model comprised 30 kNN learners, each trained on random subspaces of five features. It correctly classified 86 of 92 pathogenic (Sensitivity 93.5%, 95% CI 86.5–97%) and 37 of 43 benign (Specificity 86%, 95% CI: 72.7–93.4%) group variants within the training set. Of the variants in the test set, the model correctly predicted 19 of 23 pathogenic (Sensitivity 82.6%, 95% CI: 62.9–93%) and all 10 benign (Specificity 100%, 95% CI: 72.2–100%) group variants. VUS were scored on a 0–1 scale, with scores of 0–0.5 labeled as benign and 0.51–1 labeled as pathogenic by the classifier. (Supplementary Table S3).

### 3.3. Twenty-Two VUS Ranging in Classifier Scores Selected for Functional Analysis

**Figure 2:**
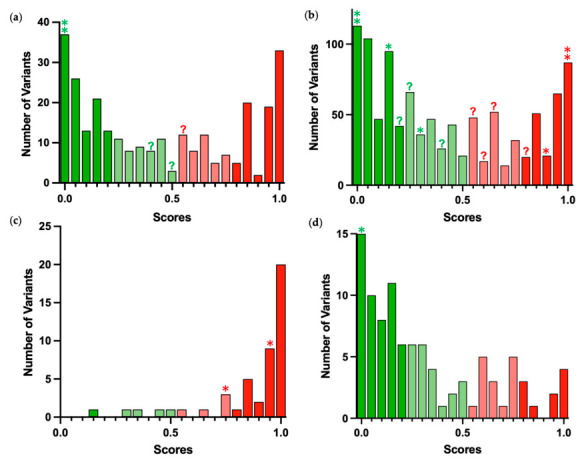
Ensemble Subspace kNN classifier score distribution and locations of selected variants for functional analysis. (**a**) Variant of uncertain significance (VUS) scores (*n* = 283) and score locations of five selected VUS. (**b**) Variants where no classification was provided scores (*n* = 764) and score locations of fourteen selected variants where no classification was provided. (**c**) Conflicting pathogenic variant scores (*n* = 47) and score locations of two selected conflicting pathogenic variants. (**d**) Conflicting benign variant scores (*n* = 97) and score location of one selected conflicting benign variant. 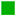 Q1 (0–0.2333); 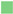 Q2 (0.2667–0.5); 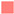 Q3 (0.5333–0.7333); 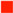 Q4 (0.7667–1); ∗ = Score locations of predicted pathogenic (red) and benign (green) variants from Q1 + Q4, with multiple ∗ in the same column denoting different variants with identical classifier scores; ? = Score locations of predicted pathogenic (red) or benign (green) variants from Q2 + Q3.

Classifier scores for VUS and variants where no classification was provided clustered towards the benign and pathogenic ends of the scale while the conflicting pathogenic and conflicting benign variants demonstrated more clustering towards pathogenic and benign ends of the scale, respectively (Figure 2). Twenty-two VUS were selected for functional assessment based on their classifier scores, including seven VUS with scores around or within the first score quartile (0–0.2333) (V1804L, V1804A, I1674V, I1674L, V1804I, I1807V, T1675S), five VUS with scores around or within the second score quartile (0.2667–0.5) (N1774H, L1839V, T1658I, L1705I, V1654L), five VUS with scores around or within the third score quartile (0.5333–0.7333) (N1774I, E1698K, Q1848K, P1749S, A1669T) and five VUS with scores around or within the fourth score quartile (0.7667–1) (R1699P, F1704S, W1837L, W1712G, F1734S). VUS in the third and fourth score quartiles were predicted as pathogenic by the classifier while VUS in the first and second score quartiles were predicted as benign by the classifier. A wildtype BRCT and two known pathogenic variants (R1699W, G1706V) were selected as controls for the VUS from the first and fourth score quartiles. A wildtype BRCT and two known pathogenic variants (R1699W, G1788V) were selected as controls for the VUS from the second and third quartiles. Additionally, an established benign variant (T1720A) was assessed along with VUS from the second and third quartiles as an additional reference for functional interpretation (Supplementary Table S4).

### 3.4. Pathogenic Controls, Benign Reference and VUS Demonstrate Varying FLAG and Phosphopeptide Signals Relative to Wildtype Control

**Figure 3:**
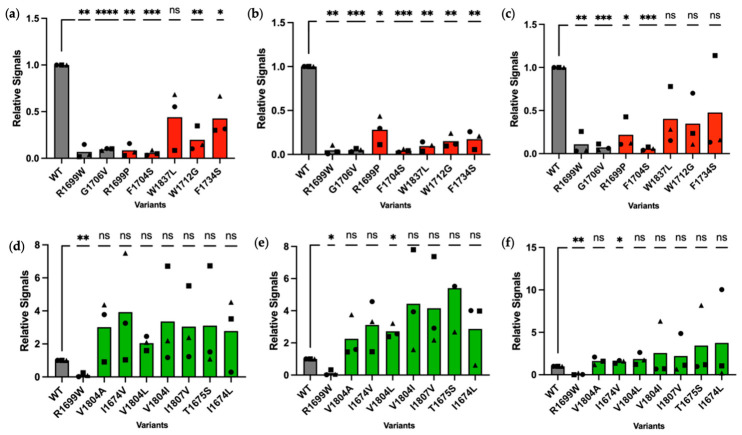
Phosphopeptide signals across variants in first and fourth score quartiles. Predicted pathogenic VUS and pathogenic controls of (**a**) Abraxas, (**b**) CtIP and (**c**) BACH1 signals relative to wildtype signals, measured in biological triplicate. Predicted benign VUS and pathogenic control (**d**) Abraxas, (**e**) CtIP and (**f**) BACH1 signals relative to wildtype signals, measured in biological triplicate. 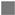 Wildtype + pathogenic controls; 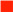 predicted pathogenic VUS; 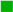 predicted benign VUS; ns = not significant; ∗ = *p* < 0.05; ∗∗ = *p* < 0.01; ∗∗∗ = *p* < 0.001; ∗∗∗∗ = *p* < 0.0001; ● = biological replicate 1; ■ = biological replicate 2; ▲ = biological replicate 3.

**Figure 4:**
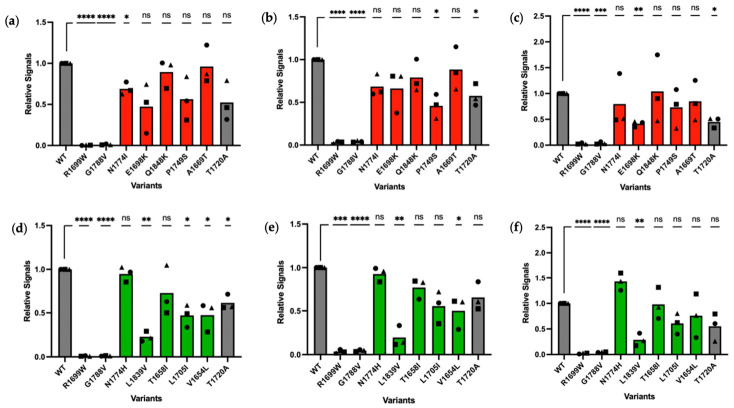
Phosphopeptide signals across variants in second and third score quartiles. Predicted pathogenic VUS, pathogenic controls and benign reference of (**a**) Abraxas, (**b**) CtIP and (**c**) BACH1 signals relative to wildtype signals, measured in biological triplicate. Predicted benign VUS, pathogenic controls and benign reference of (**d**) Abraxas, (**e**) CtIP and (**f**) BACH1 signals relative to wildtype signals, measured in biological triplicate. 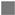 Wildtype, pathogenic controls and benign reference; 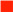 predicted pathogenic VUS; 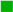 predicted benign VUS; ns = not significant; ∗ = *p* < 0.05; ∗∗ = *p* < 0.01; ∗∗∗ = *p* < 0.001; ∗∗∗∗ = *p* < 0.0001; ● = biological replicate 1; ■ = biological replicate 2; ▲ = biological replicate 3.

**Figure 5:**
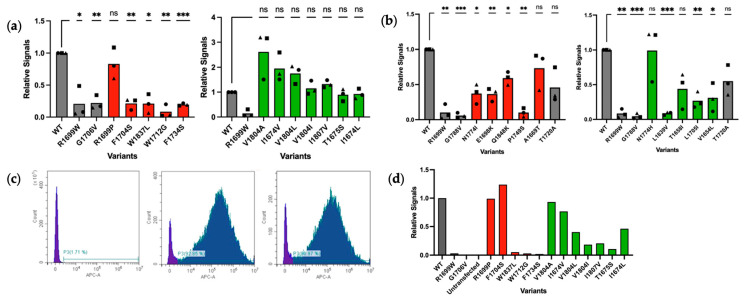
FLAG signals and investigatory flow cytometric and anti-BRCA1 immunoblot assays. (**a**) FLAG signals of the fourth score quartile predicted pathogenic VUS and pathogenic controls (**left**) and the first score quartile predicted benign VUS and pathogenic control (**right**) relative to wildtype. (**b**) FLAG signals of the third score quartile predicted pathogenic VUS, pathogenic controls and benign reference (**left**) and the second score quartile predicted benign VUS, pathogenic controls and benign reference (**right**) relative to wildtype. (**c**) Fluorescent signal intensity (APC-A) vs. cell count histograms of non-transfected (**left**), wildtype pTRE2-BRCT construct-transfected (**middle**) and pathogenic pTRE2-BRCT construct-transfected (**right**) cell samples. (**d**) FLAG signals of anti-BRCA1 co-immunoprecipitated untransfected control, VUS from the first and fourth score quartiles relative to wildtype. 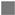 Wildtype, pathogenic controls and benign reference; 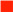 predicted pathogenic VUS; 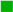 predicted benign VUS; ns = not significant; ∗ = *p* < 0.05; ∗∗ = *p* < 0.01; ∗∗∗ = *p* < 0.001; ● = biological replicate 1; ■ = biological replicate 2; ▲ = biological replicate 3.

Pathogenic controls R1699W, G1706V, G1788V and predicted benign VUS L1839V demonstrated significantly reduced signals across all phosphopeptides and FLAG relative to the wildtype (Figure 3, Figure 4 and Figure 5a,b). Predicted pathogenic VUS W1837L, W1712G, F1734S, N1774I, E1698K and P1749S and predicted benign VUS V1654L and L1705I demonstrated significantly reduced signals across only select phosphopeptides and FLAG relative to the wildtype (Figure 3, Figure 4 and Figure 5a,b). Predicted pathogenic VUS R1699P demonstrated significantly reduced signals across all phosphopeptides but no significant reductions in FLAG signals relative to the wildtype (Figure 3 and Figure 5a). Predicted pathogenic VUS F1704S demonstrated significantly reduced signals across all phosphopeptides, however anti-FLAG immunoblots demonstrated significant reductions in FLAG signals while the anti-BRCA1 immunoblot demonstrated no reductions in FLAG signals relative to the wildtype (Figure 3 and Figure 5a,d). Predicted pathogenic VUS Q1848K demonstrated no significant reductions in signals across all phosphopeptides but showed significantly reduced FLAG signals relative to the wildtype (Figure 4 and Figure 5b). Predicted benign VUS V1804A, I1674V, V1804L, V1804I, I1807V, T1675S, I1674L, N1774H and T1658I and predicted pathogenic VUS A1669T demonstrated no significant reductions in phosphopeptide or FLAG signals relative to the wildtype. (Figure 3, Figure 4 and Figure 5a,b). The benign variant T1720A demonstrated visibly reduced phosphopeptide and FLAG signals, although these were not consistently significant relative to the wildtype (Figure 4 and Figure 5b). VUS scoring in the fourth quartile demonstrated significant reductions across all or select phosphopeptides and/or FLAG signals. VUS scoring in the first quartile demonstrated no significant reductions in phosphopeptides or FLAG, while VUS scoring in the second and third quartile demonstrated varying results.

### 3.5. FLAG Signal Variability Assessed Using Flow Cytometry and Anti-BRCA1 Co-Immunoprecipitation

Relative FLAG immunoblot signals varied in intensity across VUS despite equal amounts of anti-FLAG co-immunoprecipitated cell lysate loaded in gels for each VUS (Figure 5a,b). Flow cytometry was used to compare the transfection efficiency and living/dead cell ratio between HEK293T cells transfected with wildtype and pathogenic control pTRE2-BRCT constructs modified with Cy5 fluorophore. Flow cytometric analysis showed that 1.71% of cells from the non-transfected cell sample, 92.85% of cells from the wildtype construct-transfected cell sample and 90.97% of cells from the pathogenic construct-transfected cell sample emitted fluorescence in the APC channel (Figure 5c). Both wildtype and pathogenic histograms showed similar symmetrical distributions centered around a fluorescence intensity of 10^5^. Anti-BRCA1 co-immunoprecipitation was performed on VUS from the first and fourth score quartiles to assess the efficacy of the FLAG antibody-coated magnetic beads binding to the pTRE2-BRCT construct FLAG tag compared to BRCA1 antibody-coated magnetic beads binding within the BRCT domain. The corresponding anti-BRCA1 immunoblot demonstrated FLAG signal intensity trends that generally aligned with the anti-FLAG co-immunoprecipitated immunoblots, with elevated FLAG signals observed across the wildtype control, predicted benign VUS, and the predicted pathogenic VUS, R1699P. Minimal FLAG signals were observed across the non-transfected and pathogenic controls, and predicted pathogenic VUS F1704S, W1837L, W1712G and F1734S (Figure 5d). These investigatory assays provided insight into the impact of construct transfection efficiency, transfection treatment toxicity and the binding ability of FLAG antibody-coated magnetic beads to the pTRE2-BRCT construct on the observed FLAG signal variability.

### 3.6. VUS Structural Contexts and Interactions with Phosphopeptides Visualized Through Computational Structural Modeling

**Figure 6:**
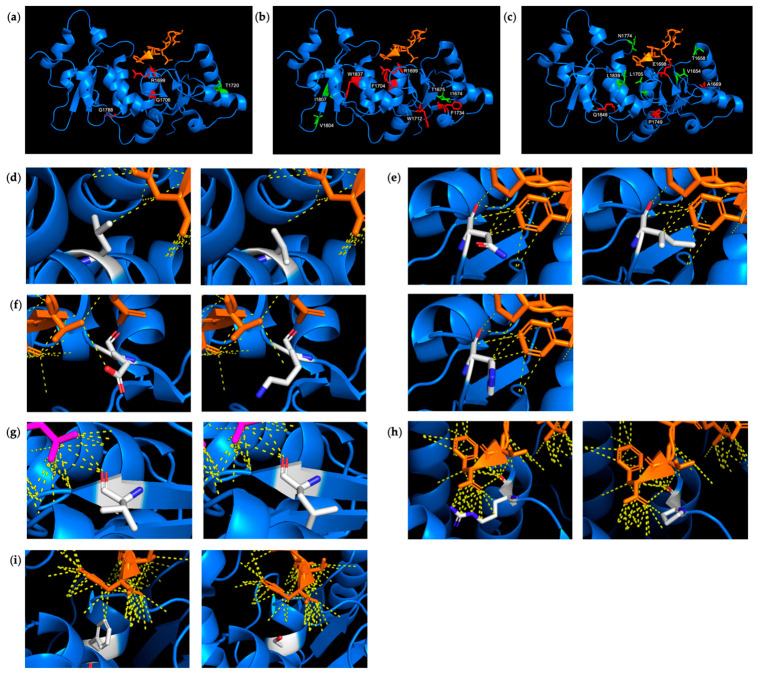
Control and VUS residues in BRCT and VUS mutagenesis. (**a**) Pathogenic controls (red) and benign reference (green) residues in BRCT (marine blue) bound to Abraxas (orange; PDB ID 4Y2G). (**b**) First score quartile predicted benign VUS residues and fourth score quartile predicted pathogenic VUS residues and in BRCT bound to Abraxas. (**c**) Second score quartile predicted benign VUS residues and third score quartile predicted pathogenic VUS residues in BRCT bound to Abraxas. (**d**) Interactions (yellow dashes) between Abraxas and wildtype L1839 (**left**) and L1839V (**right**). (**e**) Interactions between Abraxas and wildtype N1774 (**top left**), N1774I (**top right**) and N1774H (**bottom left**). (**f**) Interactions between Abraxas and wildtype E1698 (**left**) and E1698K (**right**). (**g**) Interactions between CtIP (magenta; PDB ID 1Y98) and wildtype V1654 (**left**) and V1654L (**right**). (**h**) Interactions between Abraxas and wildtype R1699 (**left**) and R1699P (**right**). (**i**) Interactions between Abraxas and wildtype F1704 (**left**) and F1704S (**right**). Residue colours for Figure 6d–i: white = main chain; navy blue = nitrogen; red = oxygen.

Controls and selected VUS were visualized in BRCA1 BRCT bound to Abraxas (PDB ID 4Y2G), BRCA1 BRCT bound to CtIP (PDB ID 1Y98) and BRCA1 BRCT bound to BACH1 (PDB ID 1T29) (Figure 6a–c). As the wildtype residues of L1839V, N1774I, N1774H, E1698K, V1654L, R1699P and F1704S directly contact one or multiple phosphopeptides, the phosphopeptide binding interactions of their corresponding VUS residues were structurally modeled on PyMOL. L1839 formed a single interchain bond with the phenylalanine anchor across all phosphopeptides. These interactions were lost when substituted with valine (Figure 6d). N1774 formed six interchain bonds with the phenylalanine anchor across all phosphopeptides. These bonds were retained when substituted with isoleucine and histidine (Figure 6e). E1698 formed a single interchain bond with a threonine residue in Abraxas and CtIP, and 14 interchain bonds with threonine, isoleucine and serine residues in BACH1. Interactions with Abraxas and CtIP were lost and BACH1 interactions were reduced to 7 when substituted with lysine (Figure 6f). V1654 formed three interchain bonds with the phosphorylated serine anchor of CtIP and BACH1. These bonds were retained when substituted with leucine (Figure 6g). R1699 formed 21 interchain bonds with the phenylalanine anchor and a threonine residue of Abraxas, 15 interchain bonds with the phenylalanine and valine residue of CtIP, and 14 interchain bonds with the phenylalanine anchor, asparagine and threonine residue of BACH1. Interactions with Abraxas were reduced to 10, interactions with CtIP were reduced to 9 and interactions with BACH1 were reduced to 9 when substituted with proline (Figure 6h). F1704 formed two interchain bonds with the phenylalanine anchor of Abraxas and a single interchain bond with the phenylalanine anchor of CtIP. These interactions were lost across Abraxas and CtIP when substituted with serine (Figure 6i). Structural modeling of VUS and the visualization of their preserved or altered number of interchain bonds with phosphopeptides provided additional information about their impact on BRCT structure and function. Structural visualization of VUS complemented both our in silico and functional evidence, as well as established structural and mechanistic properties of the BRCA1 BRCT domain, providing insight into proposed mechanisms of impact on BRCT folding and function [32,33,34,35,36,37] (Supplementary Table S5).

## 4. Discussion

In this study, we defined *BRCA1*’s RING and BRCT domains as hotspots for missense pathogenic VUS. The concentration of VUS to these domains asserts their functional importance as these VUS have been identified from populations who were eligible and selected for genetic testing due to elevated hereditary breast and ovarian cancer risk. This enrichment of VUS suggests that a proportion of RING and BRCT VUS are pathogenic but currently lack sufficient evidence for classification. Our classifier, tailored to the BRCT domain, classified BRCT VUS with ranging scores, representing varying degrees of confidence. Pull-down assays of select VUS provided functional evidence with variable concordance with model outputs. Analysis of VUS positioning within key structures and phosphopeptide interaction sites within BRCT offered additional context for the structural and functional impacts of VUS. The level of concordance among our results allowed us to establish classifier score thresholds to make calls on VUS and identify VUS that require further investigation.

The nine in silico tools selected for our classifier represent the most discriminative algorithms on known consequence *BRCA1* BRCT missense variants. They span diverse methodological frameworks, including supervised (PolyPhen2, VEST4), deep learning (EVE, MetaRNN, gMVP), supervised metapredictor (CADD hg19, BayesDel AF, ClinPred) and unsupervised meta predictor (Eigen PC) [38,39,40,41,42,43,44,45,46]. These tools primarily focus on evolutionary conservation, protein structure, protein function and variant allele frequency, capturing features relevant to BRCT domains [47]. Biochemical and sequence analyses of BRCT-bearing proteins show that BRCT domains universally function to support protein–protein interactions linked with DNA repair, recombination and cell cycle control [47]. Phylogenetic relationships show that the BRCT sequence is highly conserved among species, with several conserved residue clusters throughout [47]. The BRCT tandem architecture possesses highly conserved hydrophobic interfaces within its N and C-terminal alpha helices [47]. The association between evolutionary and structure-based features and enhanced discriminatory ability within the *BRCA1* BRCT domain suggest their elevated importance in accurately predicting VUS in this region. The integration of these tools into an Ensemble Subspace kNN classifier presented a model with the best overall performance on the validation fold and test set, highlighting their complementary strengths. Our classifier captures diverse aspects of variant pathogenicity while compensating for weaknesses in individual tools to achieve higher accuracy and robustness to noise than any single in silico tool [48]. The model’s framework, integrating 30 kNN learners trained on different random combinations of five of the nine in silico tools, reduces the generalization error arising from overfitting to small training datasets [49,50]. Our model’s accuracy across the validation fold and test set highlights its enhanced predictive ability and generalizability. Our model matched or outperformed protein language AI models AlphaMissense (86.7% validation fold, 87.9% test accuracy), ESM (82.2% validation fold, 81.8% test accuracy) and CPT-1 (67.4% validation fold, 69.7% test accuracy) [51,52,53,54]. Despite the advantages of using specific datasets to identify the most discriminative in silico features and train classifiers tailored to BRCT, the relatively small and imbalanced training and test sets present a limitation of this computational approach, as they may degrade predictor performance, increase the risk of model overfitting and reduce generalizability [55,56]. Potential external independent datasets include variants from other tandem-BRCT-bearing proteins such as BARD1, PAXIP1, LIG4, TP53BP1, MDC1, ECT2, TOPBP1, MCPH1, NBN and SLF1 [57,58]. Collectively, these proteins have 37 benign (benign and likely benign) and 3 pathogenic (pathogenic and likely pathogenic) variants documented on ClinVar as of March 2026. However, as EVE only provides predictions for a limited subset of proteins, the classifier could not evaluate variants from these proteins. Our multifactorial approach integrates functional and structural analyses of variants to provide orthogonal evidence for variant interpretation, mitigating the limitations inherent to this component of the study.

The VUS selected for functional analysis ranged in classifier scores, enabling the assessment of concordance between classifier predictions and functional results, and subsequent determination of score confidence thresholds and decision boundaries. VUS in the first and fourth score quartiles represent “high-confidence” classifications as benign or pathogenic, respectively. VUS in the second and third score quartiles represent “low-confidence” classifications as benign or pathogenic, respectively.

The controls and VUS demonstrated results consistent with four functional outcomes: (1) loss of BRCT binding ability and reduced BRCT protein levels, (2) loss of BRCT binding ability, (3) reduced BRCT protein levels, or (4) retained BRCT binding ability and BRCT protein levels. Significant reductions in phosphopeptide signal intensity relative to wildtype BRCT signify reduced phosphopeptide–BRCT domain affinity, indicating impaired phosphopeptide binding ability. Flow cytometric analysis demonstrated consistent transfection efficiencies and live cell counts across wildtype and pathogenic control pTRE2-BRCT constructs modified with Cy5 fluorophore, confirming that neither construct transfection efficiency nor transfection treatment toxicity contributed to the observed variability in FLAG signals. Anti-BRCA1 co-immunoprecipitations were conducted to evaluate whether the significant reductions in FLAG signals observed in pathogenic controls and predicted pathogenic VUS were a result of an impaired ability of the FLAG antibody-coated magnetic beads to effectively bind to the FLAG antigen due to the adjacent BRCT structure. The similar levels of FLAG observed in both anti-FLAG and anti-BRCA1 co-immunoprecipitations imply that FLAG signal intensity variability was not a result of impaired binding ability of FLAG antibody-coated magnetic beads. We propose that significantly reduced FLAG signals relative to wildtype FLAG reflects reduced steady-state BRCT protein, potentially due to protein misfolding and destabilization induced by pathogenic missense variants.

Computational structural modeling contextualized and generally supported the functional assay results. All variants directly contacting phosphopeptides and exhibited reduced bonding interactions in PyMOL analyses demonstrated functional evidence of loss-of-function (LOF) through binding reduction and/or reduced BRCT protein levels (L1839V, E1698K, V1654L, R1699P and F1704S). However, N1774I, which exhibited retained bonding interactions in PyMOL analyses, did show functional evidence of LOF. Variants positioned in or interacting with key structures within the BRCT domain, particularly its deep hydrophobic pocket, α-helices, β-sheets and linker, generally demonstrated LOF through binding reduction and/or reduced BRCT protein levels. Variants positioned in unstructured regions demonstrated less prominent functional impairment. These observations support the utility of PyMOL modeling for structural contextualization and mechanistic hypotheses. However, as PyMOL is limited by its static mutagenesis and absence of energetic refinement or molecular dynamics simulations, definitive mechanistic conclusions cannot be drawn.

Missense BRCT variants that impair phosphopeptide binding and subsequent DNA repair complex formation have been associated with increased breast and ovarian cancer risk [17]. These variants can directly impair binding by disrupting interactions with the phosphorylated serine or phenylalanine anchors or indirectly by disrupting the conformational landscape of the BRCT binding cleft [17]. As Abraxas, CtIP and BACH1-BRCT complexes are each essential for BRCA1-mediated homologous recombination, significant reductions in binding to one or more partners provide functional evidence of BRCT LOF [17]. Furthermore, several cancer-linked BRCT missense variants have shown to exhibit destabilizing effects on BRCT protein folding [59,60]. Destabilization of BRCT’s structural integrity can also serve as functional evidence for LOF as the subsequent degradation of misfolded proteins and accumulation of damaging protein aggregates dysregulate steady-state protein levels under physiological conditions [61]. Functional evidence was assigned to each VUS based on the level of significance of reductions in phosphopeptide binding and BRCT protein levels. Of note, T1720A, which was classified as benign by an expert panel, displayed visibly reduced BRCT binding ability and BRCT protein levels relative to wildtype BRCT. Our functional results show that it does not behave identically to the wildtype, suggesting that BRCT can tolerate alterations to binding and folding abilities while remaining functionally viable in vivo. Prior studies have shown that despite the traditionally high penetrance of *BRCA* genes, some pathogenic variants impart less cancer risk compared with high-penetrance protein-truncating variants [62]. Variable penetrance of variants across *BRCA1* supports the view that hereditary breast and ovarian cancer risk is better represented as a continuum rather than a binary scale. T1720A’s phosphopeptide binding and BRCT protein levels informed functional evidence thresholds. As T1720A demonstrated binding reductions across phosphopeptides at *p* < 0.05, only VUS demonstrating at least one binding reduction at *p* < 0.01 relative to the wildtype were deemed to have sufficient functional evidence for LOF. VUS with binding reductions at *p* < 0.05 were deemed to have “uncertain” functional effects. Significant reductions in BRCT protein levels also contribute to functional evidence. However, as our pull-down assay was designed to measure changes in binding ability, significant reductions in BRCT protein were not used as standalone evidence for LOF. Further studies specifically assessing the impact of VUS on BRCT folding and steady-state protein levels are required to obtain stronger functional evidence. Assays such as cycloheximide chase, pulse-chase labeling, thermal shift and proteasomal inhibition would provide further insight into protein stability and degradation kinetics, being of particularly value for the variants currently deemed to have uncertain functional effects [63,64,65,66]. VUS with significant reductions in BRCT protein levels and no significant reductions or significant reductions at *p* < 0.05 in phosphopeptide binding were deemed to have “uncertain” functional effects. Final pathogenicity predictions for each VUS were determined based on both classifier scores and functional evidence. Concordance between scores and functional effects enabled VUS to be predicted as either pathogenic or benign, while conflicting evidence or uncertain functional effects resulted in VUS pathogenicity remaining uncertain and requiring further analysis (Table 1).

Variants with uncertain pathogenicity predictions based on uncertain functional effects (Q1848K, P1749S, N1774I, L1705I, V1654L) or discordant computational and functional results (A1669T, L1839V) warrant further investigation. Variants with uncertain functional effects highlight limitations of the phosphopeptide binding assay in achieving conclusive functional evidence. Discriminating between variants that substantially impair BRCT binding ability and variants that retain sufficient activity for functional tolerance emphasizes the challenge of quantitively interpreting LOF as a non-binary phenomenon. Furthermore, as the phosphopeptide binding assay only measures one of BRCA1’s numerous mechanisms that promote genomic stability, it does not holistically assess variant impact on tumor suppressive ability. This is exemplified by A1669T, which showed no deleterious functional effects despite being predicted as pathogenic by the classifier. Although this discordance may indicate inaccuracy in the classifier prediction, it may also suggest that A1669T affects aspects of BRCA1 function not assessed through the assay. The discordance observed for L1839V, which was predicted as benign by our classifier but exhibited reduced binding ability and steady-state protein levels through our functional assays, further highlights the need for additional functional validation. HDR, cisplatin resistance and cell viability assays, while less mechanistically specific, offer valuable insight into the broader effects of variants on BRCA1-mediated genomic maintenance. Studies by Findlay et al. and Adamovich et al. provide high-throughput functional assessment of BRCA1 variants [67,68]. Findlay et al. used a survival assay in HAP1 cells to characterize the consequences of ~4000 single nucleotide variants in critical BRCA1 domains [67]. Adamovich et al. used multiplexed DNA repair assays to characterize BRCT missense variants based on HDR function and cisplatin resistance in HeLaDR-FRT cells [68]. These studies have functionally assessed most of the selected VUS in this study, providing additional insight into their functional impact on BRCA1 function (Supplementary Table S6). These assays generally corroborated the functional evidence generated by our assay, with the exception of E1698K, which exhibited LOF in our assay but was classified as functional by Findlay et al. This discordance highlights the ambiguity associated with assessing loss of BRCA1 tumor suppressive function, and how the impairment of some critical functional mechanisms may still be tolerated in vivo.

The level of concordance between classifier scores and functional results provides insight into confidence thresholds for pathogenicity prediction. VUS with scores ranging from 0–0.1333 and 0.9–1 showed complete concordance with functional results. VUS with scores ranging from 0.2333–0.5 and 0.5667–0.7667 showed general concordance with functional results with some discrepancies with functional results across some variants. Based on these results, scores between 0–0.1333 and 0.9–1 can be predicted with high confidence as benign and pathogenic, respectively. Alternatively, scores between 0.2333–0.5 and 0.5667–0.7667 can be predicted with low confidence as benign and pathogenic, respectively.

Combinations of different types of weighted evidence enable for variants to meet ACMG-AMP criteria for variant classification [20]. The Ensemble Subspace kNN provides supporting computational evidence (PP3/BP4) as per ACMG-AMP guidelines, as the model compiles multiple lines of concordant in silico evidence [20]. A novel missense change at the same position of a different pathogenic missense change constitutes moderate evidence (PM5), and is applicable to 5 variants (R1699P, W1837L P1749S, L1839V, L1705I) [20]. Our phosphopeptide-binding assay provides strong functional evidence (PS3/BS3) as per ACMG-AMP guidelines for the variants deemed LOF or functional (Table 1) [20]. Based solely on these results, R1699P, W1837L and L1839V possess sufficient evidence to be classified as likely pathogenic in accordance with ACMG-AMP rules for combining criteria to classify sequence variants (1 strong (PS3) + 1 moderate (PM5)). Moreover, N1774H, T1658I, V1804L, I1674L, V1804A, I1674V, V1804I, I1807V and T1675S possess sufficient evidence to be classified as likely benign in accordance with ACMG-AMP rules (1 strong (BS3) + 1 supporting (BP4)). Classifications for the remaining VUS require integration with other evidence types, obtained through population data, cosegregation, functional, allelic, and clinical analyses (Supplementary Table S7).

## 5. Conclusions

We defined functionally critical regions within *BRCA1* and developed methods to elucidate the effects of VUS within these regions. We aimed to improve the interpretation of BRCT missense VUS through a domain-specific classifier, phosphopeptide binding pull-down assays and computational structural modeling. We show that a classifier trained to discriminate variants in the *BRCA1* BRCT domain generally outperforms individual in silico tools and matches or outperforms AI protein language prediction models in this region. Our classifier can be applied as a method for stratifying VUS, with high-confidence VUS being most actionable and readily classifiable, and low-confidence VUS warranting further analysis. Our classifier’s performance highlights the potential utility of domain-specific models to predict the pathogenicity of variants in functional domains in *BRCA1* as well as other pleiotropic cancer susceptibility genes. The classifier and phosphopeptide binding assay generated supporting computational evidence for 4873 VUS and strong functional evidence for 17 VUS, allowing for the reclassification of 12 VUS in accordance with ACMG-AMP guidelines. Overall, this work broadens variant classification databases and will strengthen the clinical utility of genetic tests.

## Figures and Tables

**Table 1:** In silico and functional evidence for loss of BRCT function and pathogenicity predictions for select VUS. ∗ = *p* < 0.05; ∗∗ = *p* < 0.01; ∗∗∗ = *p* < 0.001; ? = ambiguous results.

Variant	Classifier Score	Abraxas	CtIP	BACH1	Protein Levels	Functional Effect
W1712G	1	∗∗	∗∗		∗∗	LOF
F1734S	1	∗	∗∗		∗∗∗	LOF
R1699P	0.9667	∗∗	∗	∗		LOF
W1837L	0.9		∗∗		∗	LOF
Q1848K	0.7667				∗	Uncertain
F1704S	0.7333	∗∗∗	∗∗∗	∗∗∗	?	LOF
P1749S	0.6333		∗		∗∗	Uncertain
N1774I	0.6	∗			∗	Uncertain
E1698K	0.5667			∗∗	∗∗	LOF
A1669T	0.5667					Functional
N1774H	0.5					Functional
T1658I	0.4333					Functional
L1839V	0.4	∗∗	∗∗	∗∗	∗∗∗	LOF
V1804L	0.3333					Functional
L1705I	0.2667	∗			∗∗	Uncertain
V1654L	0.2333	∗	∗		∗	Uncertain
I1674L	0.1333					Functional
V1804A	0					Functional
I1674V	0					Functional
V1804I	0					Functional
I1807V	0					Functional
T1675S	0					Functional

## Data Availability

Variant data analyzed in this study were obtained from ClinVar (https://www.ncbi.nlm.nih.gov/clinvar/), accessed 10 February 2024. In silico scores were obtained either through the dbNSFP database on the Ensembl Variant Effect Predictor (VEP) web interface (https://www.ensembl.org/info/docs/tools/vep/index.html), accessed 27 February 2024, or directly from individual in silico tool webpages. Structural models of *BRCA1* BRCT bound to Abraxas, CtIP and BACH1 were obtained through Protein Data Bank (PDB) (https://www.rcsb.org/). Tandem-BRCT-bearing proteins outside of BRCA1 were identified using UniProt (https://www.uniprot.org/). The data generated in this study are available within the article and its supplementary data files.

## Supplementary Materials

The following supporting information can be downloaded at: https://www.mdpi.com/article/10.3390/curroncol33060354/s1, Figure S1: Discriminatory Ability of MolecularFeaST-Ranked In Silico Tools; Table S1: In Silico Tools Ranked by MolecularFeaST; Table S2: Sequentially Trained Classifiers from MATLAB Classification Learner; Table S3: Ensemble Subspace kNN Prediction Scores and Associated Labels for VUS, Not Provided, Conflicting Pathogenic and Conflicting Benign Variants; Table S4: Selected Control, Reference and VUS ClinVar Annotations and Classifier Predictions; Table S5: Control, Reference and VUS Structural Positioning and Mechanisms of Impact; Table S6: BRCA1 Functional Assay Evidence from Torretto et al., Findlay et al. and Adamovich et al.; Table S7: Variant Classification in Accordance with ACMG-AMP Rules for Combining Weighted Evidence Criteria.

## Abbreviations

The following abbreviations are used in this manuscript:

AI	Artificial intelligence
ACMG	American College of Medical Genetics and Genomics
AMP	Association for Molecular Pathology
APC	Allophycocyanin
BME	β-mercaptoethanol
FBS	Fetal bovine serum
HBOC	Hereditary Breast and Ovarian Cancer
HDR	Homology-directed repair
HEK293T	Human Embryonic Kidney 293T
kNN	k-nearest neighbors
LB	Lysogeny broth
LOF	Loss-of-function
PBS-T	Phosphate-buffered saline + 0.1% Tween-20
SDS	Sodium dodecyl sulfate
VEP	Variant Effect Predictor
VUS	Variant of uncertain significance
