# Supplementary material for: Domain-Specific Computational, Functional and Structural Methods Enable Interpretation of BRCA1 BRCT Variants of Uncertain Significance
Source: Curr Oncol. 2026 Jun 11;33(6):354. doi: 10.3390/curroncol33060354 (PMC13298341; doi:10.3390/curroncol33060354)
Supplement: Supplementary file 1 [file curroncol-33-00354-s001.zip › Supplementary_Figure1.pdf]

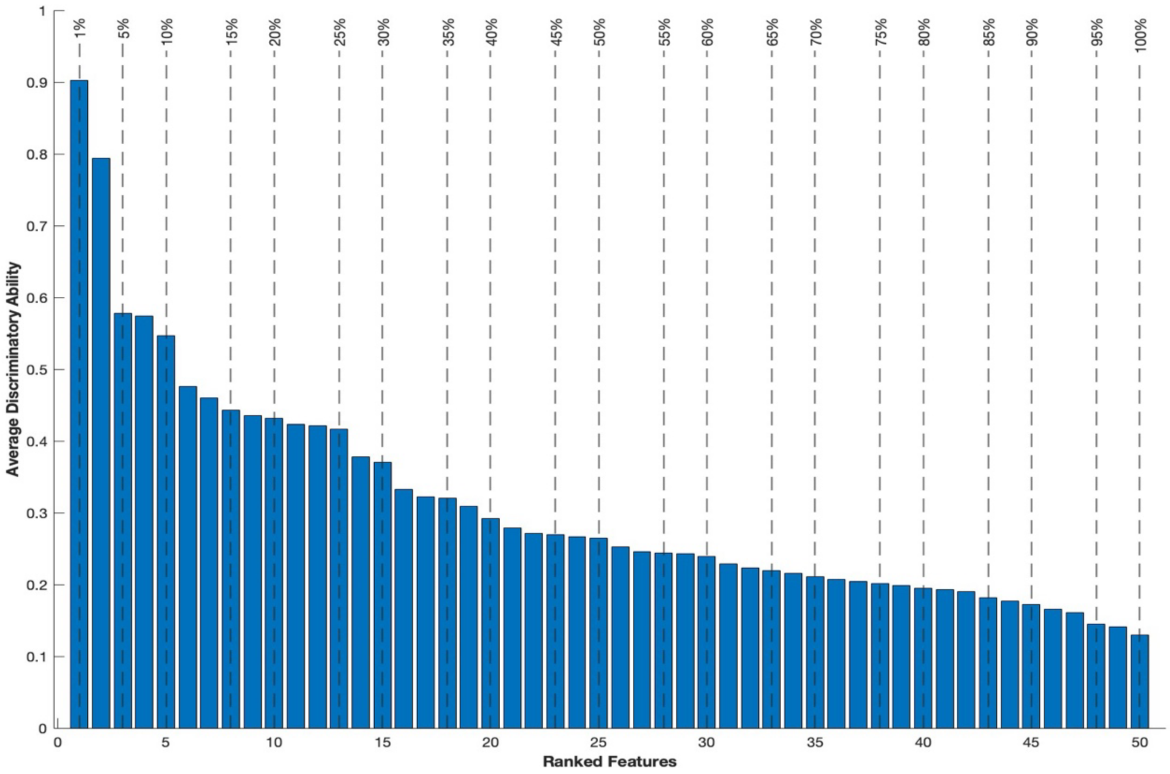

Supplementary Figure S1. Discriminatory Ability of MolecularFeaST-Ranked *In Silico* Tools. Predictive plot depicting each *in silico* tool's discriminatory ability in descending order of ranked importance. n=50.
