# Supplementary material for: Domain-Specific Computational, Functional and Structural Methods Enable Interpretation of BRCA1 BRCT Variants of Uncertain Significance
Source: Curr Oncol. 2026 Jun 11;33(6):354. doi: 10.3390/curroncol33060354 (PMC13298341; doi:10.3390/curroncol33060354)
Supplement: Supplementary file 1 [file curroncol-33-00354-s001.zip › Supplementary_Table2.pdf]

# of Tools	Classifier	Category	Accuracy on Training Set (%)	Accuracy on Test Set (%)
1	Kernel Naïve Bayes	Naïve Bayes	87.4	84.8
2	Medium kNN	kNN	90.4	84.8
3	Cosine kNN	kNN	91.1	81.8
4	Cubic kNN	kNN	91.1	84.8
5	Bagged Trees	Ensemble	91.1	78.8
6	Bagged Trees	Ensemble	92.6	81.8
7	Weighted kNN	kNN	90.4	87.9
8	Subspace kNN	Ensemble	91.1	78.8
9	Subspace kNN	Ensemble	91.1	87.9
10	Weighted kNN	kNN	90.4	84.8
11	Bagged Trees	Ensemble	90.4	87.9
12	Bagged Trees	Ensemble	85.2	84.8
13	Gaussian Naïve Bayes	Naïve Bayes	90.4	87.9

Supplementary Table S2. Sequentially Trained Classifiers from MATLAB Classification Learner. The most accurate classifiers and corresponding category on the training and test sets for each sequential combination of MolecularFeast-ranked *in silico* tools.
