# Supplementary material for: Domain-Specific Computational, Functional and Structural Methods Enable Interpretation of BRCA1 BRCT Variants of Uncertain Significance
Source: Curr Oncol. 2026 Jun 11;33(6):354. doi: 10.3390/curroncol33060354 (PMC13298341; doi:10.3390/curroncol33060354)
Supplement: Supplementary file 1 [file curroncol-33-00354-s001.zip › Supplementary_Table4.pdf]

(a)

Variant	HGVS	ClinVar Classification	Control Type
R1699Q	NM_007294.4(BRCA1):c.5096G>A (p.Arg1699Gln)	Pathogenic	Pathogenic
G1706V	NM_007294.4(BRCA1):c.5117G>T (p.Gly1706Val)	Likely Pathogenic (1 submission)	Pathogenic
G1788V	NM_007294.4(BRCA1):c.5363G>T (p.Gly1788Val)	Pathogenic	Pathogenic
T1720A	NM_007294.4(BRCA1):c.5158A>G (p.Thr1720Ala)	Benign	Benign

(b)

Variant	HGVS	ClinVar Classification	Score	Classifier Prediction
R1699P	NM_007294.4(BRCA1):c.5096G>C (p.Arg1699Pro)	Conflicting Interpretations (3 Likely Pathogenic, 3 VUS)	0.9667	Pathogenic
F1704S	NM_007294.4(BRCA1):c.5111T>C (p.Phe1704Ser)	Conflicting Interpretations (1 Likely Pathogenic, 1 VUS)	0.7333	Pathogenic
W1837L	NM_007294.4(BRCA1):c.5510G>T (p.Trp1837Leu)	Not Provided	0.9	Pathogenic
W1712G	NM_007294.4(BRCA1):c.5134T>G (p.Trp1712Gly)	Not Provided	1	Pathogenic
F1734S	NM_007294.4(BRCA1):c.5201T>C (p.Phe1734Ser)	Not Provided	1	Pathogenic
V1804L	NM_007294.4(BRCA1):c.5410G>C (p.Val1804Leu)	Not Provided	0	Benign
V1804A	NM_007294.4(BRCA1):c.5411T>C (p.Val1804Ala)	Not Provided	0	Benign
I1674V	NM_007294.4(BRCA1):c.5020A>G (p.Ile1674Val)	Not Provided	0.3333	Benign
I1674L	NM_007294.4(BRCA1):c.5020A>C (p.Ile1674Leu)	Not Provided	0.1333	Benign
V1804I	NM_007294.4(BRCA1):c.5410G>A (p.Val1804Ile)	VUS (1 submission)	0	Benign
I1807V	NM_007294.4(BRCA1):c.5419A>G (p.Ile1807Val)	Conflicting Interpretations (1 Benign, 3 VUS)	0	Benign
T1675S	NM_007294.4(BRCA1):c.5024C>G (p.Thr1675Ser)	VUS (1 submission)	0	Benign

(c)

Variant	HGVS	ClinVar Classification	Score	Classifier Prediction
N1774I	NM_007294.4(BRCA1):c.5321A>T (p.Asn1774Ile)	Not provided	0.6	Pathogenic
E1698K	NM_007294.4(BRCA1):c.5092G>A (p.Glu1698Lys)	Not provided	0.5667	Pathogenic
Q1848K	NM_007294.4(BRCA1):c.5542C>A (p.Gln1848Lys)	Not provided	0.7667	Pathogenic
P1749S	NM_007294.4(BRCA1):c.5245C>T (p.Pro1749Ser)	Not provided	0.6333	Pathogenic
A1669T	NM_007294.4(BRCA1):c.5005G>A (p.Ala1669Thr)	VUS	0.5667	Pathogenic
N1774H	NM_007294.4(BRCA1):c.5320A>C (p.Asn1774His)	VUS	0.5	Benign
L1839V	NM_007294.4(BRCA1):c.5515T>G (p.Leu1839Val)	Not provided	0.4	Benign
T1658I	NM_007294.4(BRCA1):c.4973C>T (p.Thr1658Ile)	VUS	0.4333	Benign
L1705I	NM_007294.4(BRCA1):c.5113C>A (p.Leu1705Ile)	Not provided	0.2667	Benign
V1654L	NM_007294.4(BRCA1):c.4960G>C (p.Val1654Leu)	Not provided	0.2333	Benign

Supplementary Table S4. Selected Control, Reference and VUS ClinVar Annotations and Classifier Predictions. (a) Three pathogenic controls and one benign variant reference. **(b)** Five high confidence predicted pathogenic VUS and seven high confidence predicted benign VUS. **(c)** Five low confidence predicted pathogenic VUS and five low confidence predicted benign VUS.
