# Supplementary material for: Domain-Specific Computational, Functional and Structural Methods Enable Interpretation of BRCA1 BRCT Variants of Uncertain Significance
Source: Curr Oncol. 2026 Jun 11;33(6):354. doi: 10.3390/curroncol33060354 (PMC13298341; doi:10.3390/curroncol33060354)
Supplement: Supplementary file 1 [file curroncol-33-00354-s001.zip › Supplementary_Table5.pdf]

(a)

Variant	Structural Positioning	Established Mechanisms of Impact
**R1699W**	pSer-X-X-Phe motif interaction interface	Causes conformational instability through the loss of salt-bridging interactions and significant binding reductions to the phosphopeptides' phenylalanine anchors [17,32,33,59].
**G1706V**	BRCT1 α2	Diminishes hydrogen bonding networks within the BRCT hydrophobic binding cleft [34].
**G1788V**	BRCT2 α1′	Highly destabilizes BRCT's hydrophobic core by disrupting van der Walls contacts between constituting α-helices and β-sheets [59].
**T1720**	BRCT1 a3	Contributes to BRCT2's deep hydrophobic pocket but makes no direct contact with the phosphopeptide binding motif [32,33]. Structural location and residue substitution from polar threonine to nonpolar alanine potentially disrupts hydrogen bonds that contribute to the binding cleft. Does not behave identically to wildtype BRCT, suggesting that BRCT can tolerate alterations to folding and binding abilities while remaining functionally viable *in vivo*.

(b)

Variant	Structural Positioning	Proposed Mechanisms of Impact
**V1804L**	-	Localizes outside of BRCT's phosphopeptide binding cleft and conserved structures, unlikely to impact binding or protein folding [34].
**V1804A**	-	Localizes outside of BRCT's phosphopeptide binding cleft and conserved structures, unlikely to impact binding or protein folding [34].
**I1674V**	BRCT1 β2	BRCT1 β2 contributes to overall BRCT architecture through hydrophobic interactions with neighbouring structures [32,33,35]. Although I1674 may participate in these interactions, substitution between biochemically similar residues (nonpolar to nonpolar) is unlikely to cause aberrant folding.
**I1674L**	BRCT1 β2	BRCT1 β2 contributes to overall BRCT architecture through hydrophobic interactions with neighbouring structures [32,33,35]. Although I1674 may participate in these interactions, substitution between biochemically similar residues (nonpolar to nonpolar) is unlikely to cause aberrant folding.
**V1804I**	-	Localizes outside of BRCT's phosphopeptide binding cleft and conserved structures, unlikely to impact binding or protein folding [34].
**I1807V**	BRCT2 β3′	BRCT β3′ contributes to overall BRCT architecture through hydrophobic interactions with neighbouring structures [32,33,35]. Although I1807 may participate in these interactions, substitution between biochemically similar residues (nonpolar to nonpolar) is unlikely to cause aberrant folding.
**T1675S**	BRCT1 β2	BRCT1 β2 contributes to overall BRCT architecture through hydrophobic interactions with neighbouring structures [32,33,35]. Although T1675 may participate in these interactions, substitution between biochemically similar residues (polar to polar) is unlikely to cause aberrant folding.
**N1774H**	pSer-X-X-Phe motif interaction interface	Structural visualization shows no impairment to interactions across all phosphopeptides. The substitution from polar asparagine to polar histidine is unlikely to significantly alter binding interactions and conformational stability within the binding cleft.
**L1839V**	pSer-X-X-Phe motif interaction interface, BRCT α3′	BRCT2 α3′ forms van der Walls contacts within BRCT's hydrophobic core. Component of BRCT2's deep hydrophobic pocket within the motif interaction interface, interacting with the phenylalanine anchor across phosphopeptides [32,33]. Structural modeling of L1839V showed impairment of all interactions across phosphopeptides.
**T1658I**	-	Localizes outside of BRCT's phosphopeptide binding cleft and conserved structures, unlikely to impact binding or protein folding [34].
**L1705I**	BRCT1 α2	BRCT1 α2 is a key structural component of BRCT's hydrophobic core and binding cleft [32,33]. Despite L1705I representing structurally and biochemically conservative residue changes, its positioning within a critical structural region may underlie observed reduced binding ability and protein levels.
**V1654L**	BRCT1 β1	Structural visualization shows interactions with the phosphorylated serine anchor of CtIP and BACH1. Although structural modeling shows no impairment of interactions and structurally and biochemically conservative residue change, its positioning within critical structures and binding sites may underlie observed reduced binding ability and protein levels.
**N1774I**	pSer-X-X-Phe motif interaction interface	Structural visualization shows similar residue orientation and retains interactions with the phenylalanine anchor relative to the wildtype residue. A substitution from a polar to nonpolar residue may affect hydrogen bonds with phosphopeptides as well as conformational stability within the binding cleft.

**E1698K**	-	Localizes within the BRCT hydrophobic binding cleft, interacting with all phosphopeptides [32,33]. Structural modeling of E1698K shows complete loss of interactions with Abraxas and CtIP and reduced BACH1 interchain bonds by half. Interactions with multiple residues of BACH1 and E1698K's significant reduction in binding to only BACH1 in functional studies indicate the residue's elevated importance in BACH1 binding.
**Q1848K**	-	Localizes to a non-structured region near the end of BRCT2, however structural modeling reveals hydrogen bonds with BRCT α3 [32,33]. The introduction of a charged lysine residue may disrupt these interactions with BRCT α3, potentially altering its structural orientation and subsequent conformation within BRCT.
**P1749S**	αL	Locates within the conserved linker between BRCT1 and BRCT2 [32,33]. Substitution from proline to a polar serine potentially disrupts αL coiling and overall BRCT folding [36]. Variants within αL may alter the positioning of BRCT1 and BRCT2 in forming the binding cleft, potentially reducing binding ability [37].
**A1699T**	BRCT1 α1	BRCT1 α1 contributes to overall BRCT architecture through hydrophobic interactions with neighbouring structures [32,33]. Although substitution from nonpolar alanine to polar threonine suggests structural implications, both residues are small and compactible within helix formation. Unlikely to severely disrupt BRCT1 α1 conformation and BRCT folding.
**R1699P**	pSer-X-X-Phe motif interaction interface	Directly interacts with the phenylalanine anchor and adjacent residues across all phosphopeptides [32,33]. Structural modeling of R1699P shows impairment of interactions with phenylalanine anchor across phosphopeptides. Proline substitution unlikely to impose severe backbone strain that would disrupt BRCT folding.
**F1704S**	pSer-X-X-Phe motif interaction interface, BRCT1 α2	BRCT1 α2 is a key component of BRCT hydrophobic binding cleft. F1704 interacts with the phenylalanine anchor across all phosphopeptides [32,33]. Structural modeling of F1704S shows impairment of interactions with phenylalanine anchor across all phosphopeptides. Substitution from aromatic nonpolar phenylalanine to polar serine potentially disrupts key hydrophobic interactions.<br>F1704S may impair protein folding to an extent that hinders FLAG tag recognition, but not severely enough to induce protein degradation.
**W1837L**	BRCT2 α3′	BRCT2 α3′ key component of BRCT's hydrophobic binding cleft [47,48]. Substitution from flexible tryptophan to compact leucine potentially destabilizes key hydrophobic interactions and binding cleft formation [32,33,60].
**W1712G**	BRCT1 β4	BRCT1 β4 contributes to a BRCT surface groove on the opposite face of its binding cleft. Groove conformational changes weaken partner protein binding critical for BRCA1 transactivation activity [34]. Aromatic tryptophan to nonpolar glycine substitution potentially disrupts groove stability.
**F1734S**	Unnamed alpha helical structure (aa 1731-1734)	Unnamed α-helical structure proximal to BRCT1 α3 and BRCT1 β4 [32,33]. Substitution from aromatic phenylalanine to the polar serine potentially disrupts hydrophobic interactions that contribute to the stability of the BRCT hydrophobic core.

Supplementary Table S5. Control, Reference and VUS Structural Positioning and Mechanisms of Impact. (a) Established mechanisms of impact of three pathogenic controls. **(b)** Proposed mechanisms of impact of twenty-two VUS and one benign variant reference.
