# Supplementary material for: Domain-Specific Computational, Functional and Structural Methods Enable Interpretation of BRCA1 BRCT Variants of Uncertain Significance
Source: Curr Oncol. 2026 Jun 11;33(6):354. doi: 10.3390/curroncol33060354 (PMC13298341; doi:10.3390/curroncol33060354)
Supplement: Supplementary file 1 [file curroncol-33-00354-s001.zip › Supplementary_Table6 .pdf]

Variant	Phosphopeptide Binding (Torretto et al.)	Cell Viability (Findlay et al.)	HDR (Adamovich et al.)	Cisplatin Resistance (Adamovich et al.)
W1712G	LOF	LOF	LOF	LOF
F1734S	LOF	LOF	LOF	LOF
R1699P	LOF	LOF	LOF	LOF
W1837L	LOF	LOF	-	-
Q1848K	-	Functional	-	Functional
F1704S	LOF	LOF	LOF	LOF
P1749S	-	LOF	-	Functional
N1774I	-	Functional	-	-
E1698K	LOF	Functional	-	-
A1669T	Functional	Functional	Functional	-
N1774H	Functional	Functional	Functional	Functional
T1658I	Functional	-	Functional	-
L1839V	LOF	LOF	-	LOF
V1804L	Functional	Functional	Functional	Functional
L1705I	-	Functional	Functional	Functional
V1654L	-	Functional	-	-
I1674L	Functional	Functional	Functional	Functional
V1804A	Functional	Functional	Functional	Functional
I1674V	Functional	Functional	Functional	Functional
V1804I	Functional	Functional	-	-
I1807V	Functional	Functional	-	-
T1675S	Functional	Functional	Functional	Functional

Supplementary Table S6. *BRCA1* Functional Assay Evidence from Torretto et al., Findlay et al. and Adamovich et al.

- = Not available
