# Supplementary material for: Domain-Specific Computational, Functional and Structural Methods Enable Interpretation of BRCA1 BRCT Variants of Uncertain Significance
Source: Curr Oncol. 2026 Jun 11;33(6):354. doi: 10.3390/curroncol33060354 (PMC13298341; doi:10.3390/curroncol33060354)
Supplement: Supplementary file 1 [file curroncol-33-00354-s001.zip › Supplementary_Table7.pdf]

Variant	PS3	PM5	PP3	BS3	BP4	Classification
W1712G	✓		✓			VUS
F1734S	✓		✓			VUS
R1699P	✓	✓	✓			Likely Pathogenic
W1837L	✓	✓	✓			Likely Pathogenic
Q1848K			✓			VUS
F1704S	✓		✓			VUS
P1749S		✓	✓			VUS
N1774I			✓			VUS
E1698K	✓		✓			VUS
A1669T			✓	✓		VUS
N1774H				✓	✓	Likely Benign
T1658I				✓	✓	Likely Benign
L1839V	✓	✓			✓	Likely Pathogenic
V1804L				✓	✓	Likely Benign
L1705I					✓	VUS
V1654L					✓	VUS
I1674L				✓	✓	VUS
V1804A				✓	✓	Likely Benign
I1674V				✓	✓	Likely Benign
V1804I				✓	✓	Likely Benign
I1807V				✓	✓	Likely Benign
T1675S				✓	✓	Likely Benign

Supplementary Table S7. Variant Classification in Accordance with ACMG-AMP Rules for Combining Weighted Evidence Criteria. Classifications based only on strong functional, moderate locational and supporting computational evidence curated in this study.

PS3: Well-established *in vitro* or *in vivo* functional studies supportive of a damaging effect on gene
PM5: Novel missense change at an amino acid residue where a different missense change determined to be pathogenic has been seen before
PP3: Multiple lines of computational evidence supporting a deleterious effect on gene
BS3: Well-established *in vitro* or *in vivo* functional studies showing no damaging effect on protein function or splicing
BP4: Multiple lines of computational evidence suggest no impact on gene
